# An Experimental and Systematic Insight into the Temperature Sensitivity for a 0.15-µm Gate-Length HEMT Based on the GaN Technology

**DOI:** 10.3390/mi12050549

**Published:** 2021-05-12

**Authors:** Mohammad Abdul Alim, Christophe Gaquiere, Giovanni Crupi

**Affiliations:** 1 Department of Electrical and Electronic Engineering, University of Chittagong, Chittagong 4331, Bangladesh; mohammadabdulalim@cu.ac.bd; 2 Institute of Electronic, Microelectronic and Nanotechnology (IEMN), The University of Lille, F-59000 Lille, France; christophe.gaquiere@iemn.univ-lille1.fr; 3 Department of Biomedical and Dental Sciences and Morphofunctional Imaging, University of Messina, 98125 Messina, Italy

**Keywords:** gallium nitride (GaN), high electron-mobility transistor (HEMT), equivalent-circuit modeling, microwave frequency, scattering-parameter measurements, temperature

## Abstract

Presently, growing attention is being given to the analysis of the impact of the ambient temperature on the GaN HEMT performance. The present article is aimed at investigating both DC and microwave characteristics of a GaN-based HEMT versus the ambient temperature using measured data, an equivalent-circuit model, and a sensitivity-based analysis. The tested device is a 0.15-μm ultra-short gate-length AlGaN/GaN HEMT with a gate width of 200 μm. The interdigitated layout of this device is based on four fingers, each with a length of 50 μm. The scattering parameters are measured from 45 MHz to 50 GHz with the ambient temperature varied from −40 °C to 150 °C. A systematic study of the temperature-dependent performance is carried out by means of a sensitivity-based analysis. The achieved findings show that by the heating the transistor, the DC and microwave performance are degraded, due to the degradation in the electron transport properties.

## 1. Introduction

As well-known, high electron-mobility transistors (HEMTs) based on the aluminum gallium nitride/gallium nitride (AlGaN/GaN) heterojunction are outstanding candidates for high-frequency, high-power, and high-temperature applications, owing to the unique physical properties of the GaN material. Throughout the years, many studies have been dedicated to the investigation of how the temperature impacts the performance of GaN-based HEMT devices. To this end, both electro-thermal simulations [1,2,3,4,5,6] and measurement-based analysis [7,8,9,10,11,12,13,14,15,16,17,18,19,20,21,22,23,24,25,26] have been developed. Although the electro-thermal device simulation is undoubtedly a very powerful and costless tool to deeply understand the underlying physics behind the operation of the transistor in order to improve the device fabrication, the measurement-based investigation is a step of crucial importance for achieving a reliable validation of a transistor technology prior to its use in real applications. Typically, measurements are coupled with the extraction of a small-signal equivalent-circuit model, which can be used as cornerstone for building both large-signal [27,28,29] and noise [30,31,32] transistor models that are essential for a successful design of microwave high-power [33,34,35,36] and low-noise amplifiers [36,37,38]. Compared to the effective modeling approach based on using artificial neural networks (ANNs) [39,40], the equivalent-circuit model allows a physically meaningful description [41,42,43], thereby enabling development of a sensitivity-based investigation.

To gain a comprehensive insight, the present article focuses on the impact of the ambient temperature (T_a_) on the behavior of an on-wafer GaN HEMT using DC and microwave measurements coupled with a small-signal equivalent-circuit model and a sensitivity-based analysis. The device under test (DUT) is an ultra-short gate-length HEMT based on an AlGaN/GaN heterojunction grown on a silicon carbide (SiC) substrate. The DUT has a gate length of 0.15 μm and a gate width of 200 μm. The interdigitated layout consists of four fingers, each being 50-μm long. The DC characteristics and the scattering parameters from 45 MHz to 50 GHz are measured at nine different ambient temperature conditions by both cooling and heating the device, spanning the −40 °C to 150 °C temperature range. The measured data are used for equivalent-circuit extraction and sensitivity-based analysis, enabling one to assess the impact of the variation in the ambient temperature on the transistor performance. Basically, the main goal of this work is to extend the results of a previous article focused on the same DUT [15] by developing a sensitivity-based analysis, thus enabling a quantitative and systematic investigation of the effects of changes in the ambient temperature on the DC and microwave characteristics. Nevertheless, it should be pointed out that the obtained results are not of general validity, as they may strongly depend on the selected device and operating bias condition.

The paper is structured with the following sections. Section 2 describes the DUT and the experimental characterization, Section 3 reports and discusses the achieved findings, and Section 4 presents the conclusions.

## 2. Device under Test and Experimental Details

**Figure 1:**
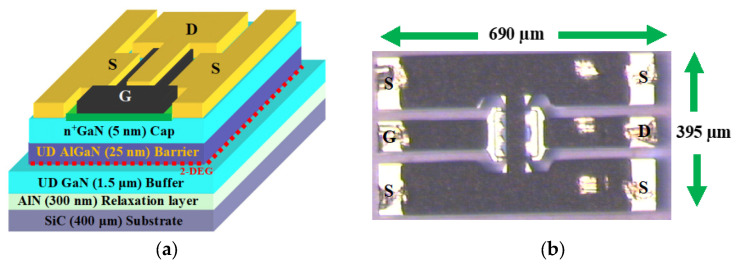
(**a**) Schematic drawing of the epitaxial structure and (**b**) photograph of the tested 0.15 μm × (4 × 50) μm GaN HEMT.

The metal organic chemical vapor deposition (MOCVD) technique is used to grow the Al_0.253_Ga_0.747_N/GaN heterostructure on a 400-μm-thick SiC substrate. The schematic cross-sectional view and the photograph of the tested GaN HEMT are illustrated in Figure 1. The epitaxial layer structure of the device is made up of a 25-nm-thick undoped (UD) AlGaN barrier and a 1.5-μm-thick UD GaN buffer layer. A 300-nm-thick graded AlN relaxation layer was grown between the GaN buffer and the SiC substrate. The device was capped with a 5-nm-tick n+-GaN layer. The evaporation process was employed to create the source and drain ohmic contacts (Ti/Al/Ni/Au with thicknesses of 12/200/40/100 nm, respectively) and followed by 30 s of thermal annealing at 900 °C. The Schottky mushroom-shaped gate was formed through Pt/Ti/Pt/Au evaporation and the subsequent lift-off process. Finally, a Si_3_N_4_ layer with a thickness of 240 nm was deposited to passivate the device. The gate length of the tested GaN device is 0.15 μm. The interdigitated architecture of the device is based on the parallel connection of four 50-μm long fingers, resulting in a total gate width of 200 μm. The source-to-gate distance (L_SG_) and the gate-to-drain distance (L_GD_) are 1 μm and 2.85 μm, respectively. The DUT was fabricated at the University of Lille, France.

**Figure 2:**
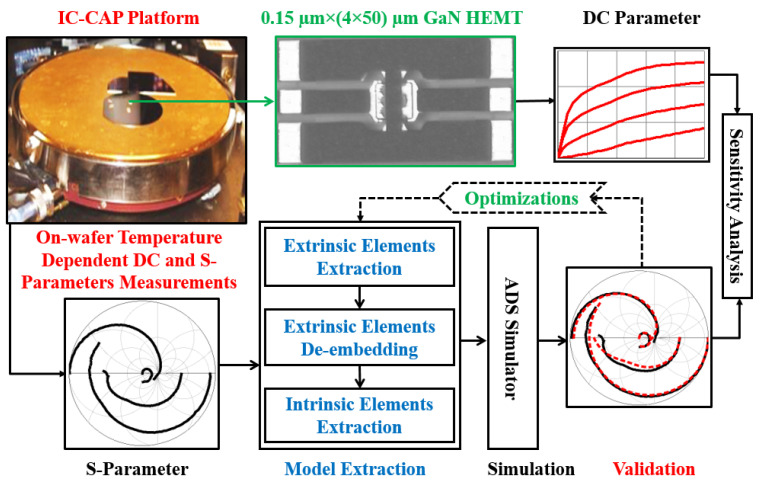
The flow diagram of the measurement process, model extraction, and sensitivity-based analysis for the tested on-wafer GaN HEMT.

The microwave experiments consist of DC and S-parameters measured from 45 MHz to 50 GHz at nine different ambient temperatures: −40 °C, −25 °C, 0 °C, 25 °C, 50 °C, 75 °C, 100 °C, 125 °C, and 150 °C. The analysis is performed using the DC characteristics and the S-parameters at a bias point in the saturation region: V_ds_ = 15 and V_gs_ = −5 V. The device parameters were measured with a thermal probe station connected to an HP8510C vector network analyzer (VNA) and with the aid of commercially available software to guarantee that the data are free of human error. The DC and frequency-dependent measurements were performed at each temperature after the sample reached uniform steady-state temperature. Figure 2 shows the measurement process, model extraction, and sensitivity-based analysis.

## 3. Experimental Results and Systematic Analysis

The systematic sensitivity-based analysis at the selected bias voltages is accomplished using the dimensionless relative sensitivity of each parameter (*RSP*) with respect to *T_a_*, which is calculated by normalizing the relative change in *P* to the relative change in *T_a_*:

(1) $$RSP=\frac{\Delta P}{P_{0}}\frac{T_{a0}}{\Delta T_{a}}=\frac{\left(P-P_{0}\right)}{P_{0}}\frac{T_{a0}}{\left(T_{a}-T_{a0}\right)}$$

where *P*_0_ is the value of the selected parameter *P* at the reference temperature (*T_a_*_0_) of 25 °C.

The remainder of this section is divided into two subsections: the first part is focused on the impact of the ambient temperature on the DC characteristics, whereas the second part is dedicated to the effects of the variations in the ambient temperature on the microwave performance.

### 3.1. Sensitivity-Based Analysis of DC Characteristics

**Figure 3:**
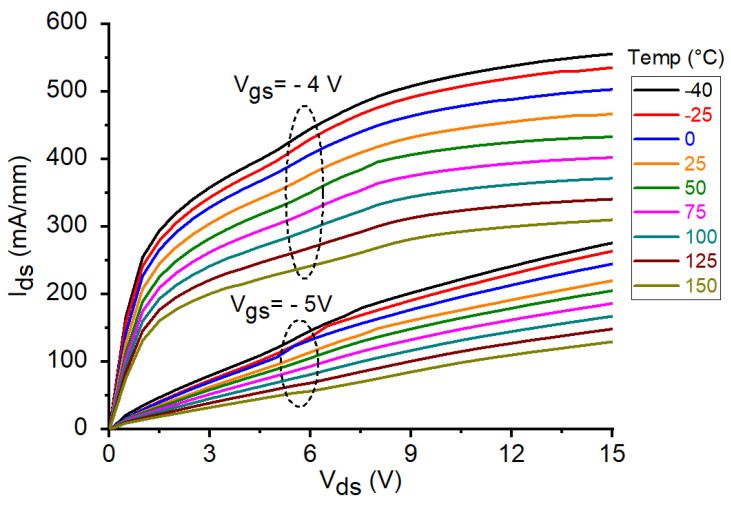
DC output characteristics of the studied GaN HEMT at V_gs_ = −4 V and −5 V under different temperature conditions.

**Figure 4:**
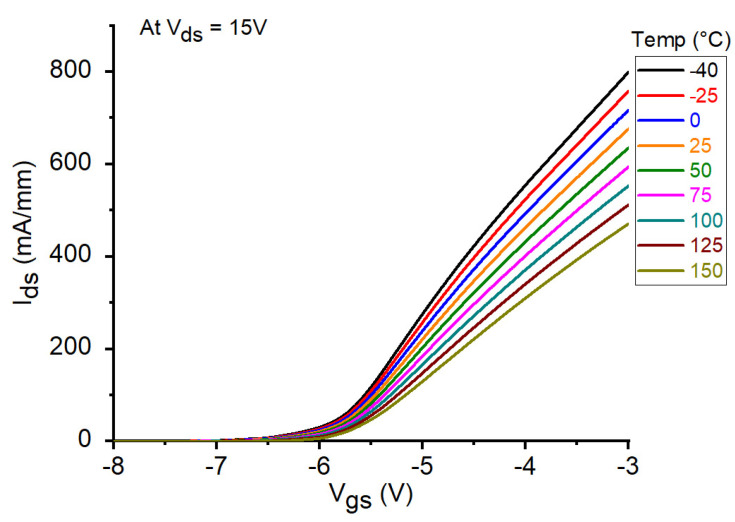
DC transcharacteristics of the studied GaN HEMT at V_ds_ = 15 V under different temperature conditions.

**Figure 5:**
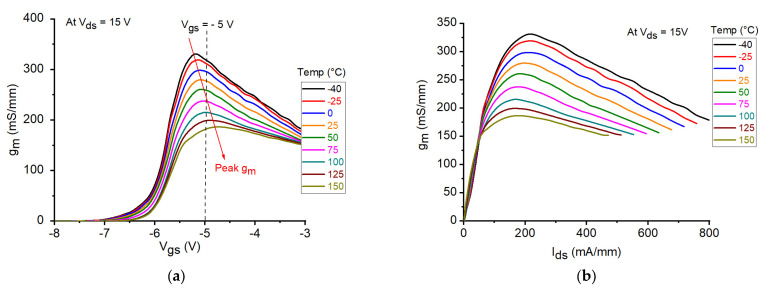
DC transconductance of the studied GaN HEMT at V_ds_ = 15 V under different temperature conditions. The data are reported as a function of (**a**) V_gs_ and (**b**) I_ds_.

**Figure 6:**
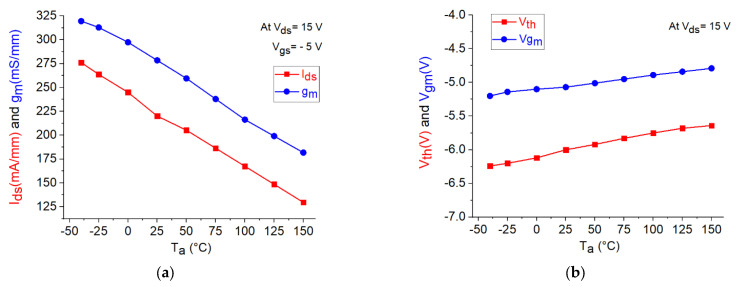
Temperature dependence of the DC parameters of the studied GaN HEMT: (**a**) I_ds_ and g_m_ at V_ds_ = 15 V and V_gs_ = −5 V; (**b**) V_th_ and V_gm_ (i.e.,V_gs_ at which g_m_ shows its peak value) at V_ds_ = 15 V.

The DC output characteristics for the tested GaN HEMT at V_gs_ = −4 V and −5 V under different temperature conditions are illustrated in Figure 3. As can be clearly observed, I_ds_ is considerably reduced with increasing temperature. This might be attributed to the degradation in the carrier transport properties as a consequence of the enhancement of the phonon-scattering processes at higher temperatures. Analogously, the reduction in I_ds_ at higher temperatures can be observed by plotting the DC transcharacteristics of the studied device at V_ds_ = 15 V (see Figure 4). Similar fashion of degradation can be seen in the transconductance by plotting the g_m_-V_gs_ curves at V_ds_ = 15 V (see Figure 5a). As a matter of the fact, by heating the device, the transconductance is significantly reduced. However, it should be underlined that a higher temperature leads to a wider and flatter curve of g_m_ versus V_gs_, thus implying a better linearity. Over the years, many studies have been devoted at improving the flatness of g_m_ versus V_gs_, in order to yield to an improved transistor linearity and then to a more linear power amplifier [44,45]. For the sake of completeness, the behavior of g_m_ is plotted also as a function of I_ds_ (see Figure 5b). At the selected bias point: V_ds_ = 15 V and V_gs_ = −5 V, both I_ds_ and g_m_ are significantly degraded when the temperature is raised, as illustrated in Figure 6a. The interesting feature found in the g_m_−V_gs_ curves of Figure 5a is that, by heating the device, the peak value of g_m_ is not only greatly reduced but also shifted toward less negative values of V_gs_. As shown in Figure 6b, the value of V_gs_ at which the peak in g_m_ occurs (V_gm_) is increased from −5.2 V at −40 °C to −4.8 V at 150 °C. It is worth noting that also the threshold voltage (V_th_) shifts toward less negative values at higher T_a_. As illustrated in Figure 6b, V_th_ is increased from −6.24 V at −40 °C to −5.64 V at 150 °C.

**Figure 7:**
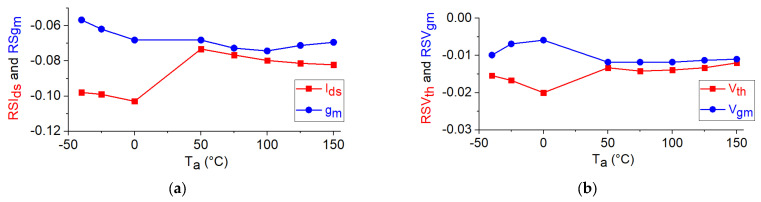
Temperature dependence of the relative sensitivities of the DC parameters of the studied GaN HEMT: (**a**) RSI_ds_ and RSg_m_ at V_ds_ = 15 V and V_gs_ = −5 V; (**b**) RSV_th_ and RSV_gm_ at V_ds_ = 15 V.

Using Equation (1), the relative sensitivities of I_ds_, g_m_, V_gm_, and V_th_ with respect to T_a_ are calculated and reported in Figure 7. As can be observed, RSI_ds_, RSg_m_, RSV_th_, and RSV_gm_ are negative for the studied device, as a consequence of the fact that an increase in T_a_ leads to a reduction in the values of I_ds,_ g_m_, V_th_, and V_gm_.

### 3.2. Sensitivity-Based Analysis of Small-Signal Parameters and RF Figures of Merit

**Figure 8:**
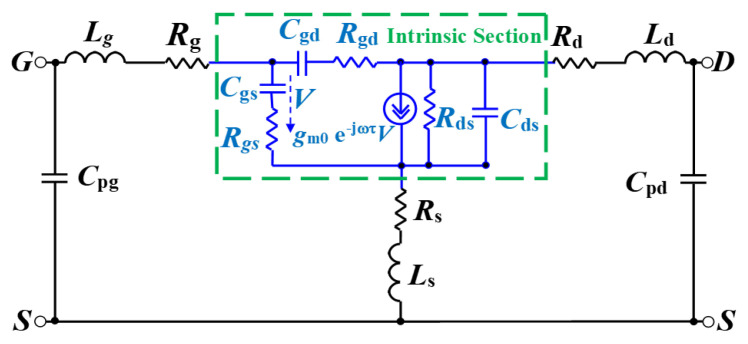
The equivalent-circuit model for the GaN HEMT under investigation.

**Figure 9:**
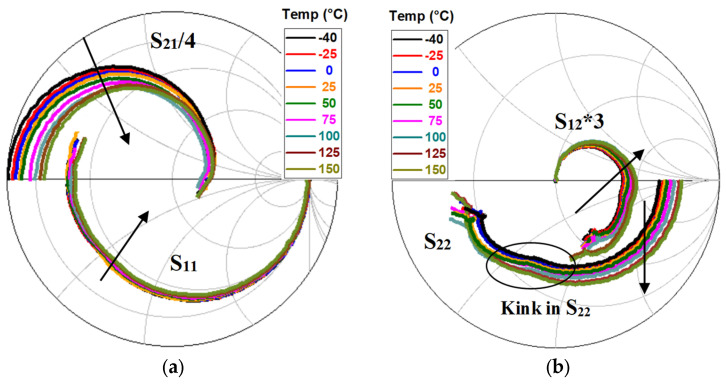
Measured S-parameters of the studied GaN HEMT at V_ds_ = 15 V and V_gs_ = −5 V under different temperature conditions: (**a**) S_11_, S_21_, (**b**) S_12_, and S_22_.

**Figure 10:**
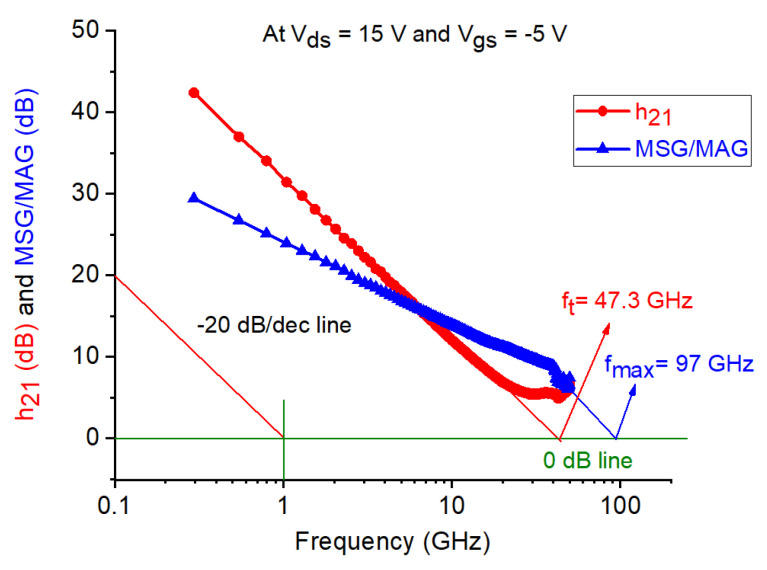
Behavior of the magnitude of the h_21_ and MAG/MSG versus the frequency of the studied GaN HEMT at V_ds_ = 15 V, V_gs_ = −5 V, and T_a_ = 25 °C.

The equivalent-circuit model in Figure 8 was used to model the measured S-parameters of the studied device. The equivalent-circuit parameters (ECPs) were extracted as described in [15], using the well-known “cold” pinch-off approach that has been widely and successfully applied to the GaN technology over the years [46,47,48,49,50]. The effect of T_a_ on the measured S-parameters at the selected bias point is shown in Figure 9. It should be highlighted that as the carrier transport properties deteriorate with increasing T_a_, the low-frequency magnitude of S_21_ is reduced. This is in line with the degradation of the DC g_m_ at higher T_a_ (see Figure 5). As can be observed, the tested device is affected by the kink effect in S_22_. As well-known, the GaN HEMT technology is prone to be affected by this phenomenon, owing to the relatively high transconductance [51,52,53,54]. In accordance with this, the observed kink effect in S_22_ is more pronounced at lower T_a_, due to the higher g_m_. The DC parameters, ECPs, intrinsic input and feedback time constants (i.e., τ_gs_ = R_gs_C_gs_ and τ_gd_ = R_gd_C_gd_), the unity current gain cut-off frequency (f_t_), and the maximum frequency of oscillation (f_max_) are reported at 25 °C in Table 1. The three intrinsic time constants (τ_m_, τ_gs_, and τ_gd_), which emerge from the inertia of the intrinsic transistor in reacting to rapid signal changes, are meant to represent the intrinsic non-quasi-static (NQS) effects, which play a more significant role at higher frequencies. The values of f_t_ and f_max_ are obtained from the frequency-dependent behavior of the measured short-circuit current gain (h_21_) and maximum stable/available gain (MSG/MAG), respectively (see Figure 10).

**Figure 11:**
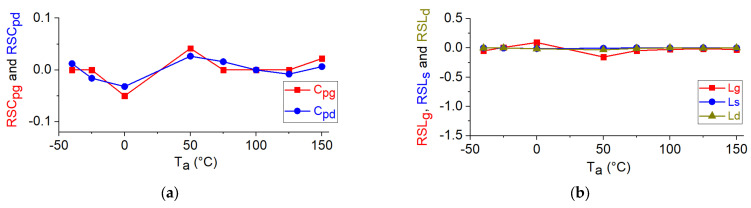

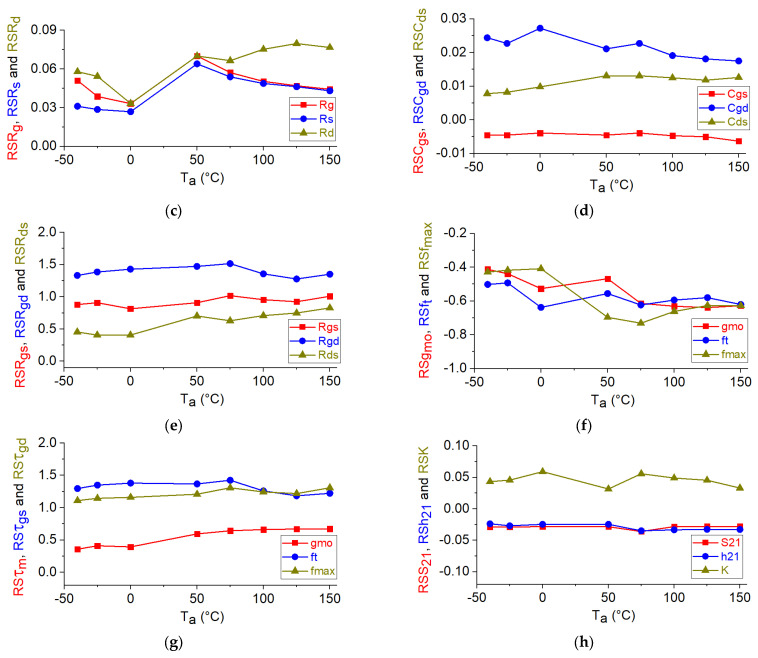
The relative sensitivities of the analyzed parameters versus ambient temperature for the studied GaN HEMT: (**a**) RSC_pg_ and RSC_pd_; (**b**) RSL_g_, RSL_s_, and RSL_d_; (**c**) RSR_g_, RSR_s_, and RSR_d_; (**d**) RSC_gs_, RSC_gd_, and RSC_ds_; (**e**) RSR_gs_, RSR_gd_, and RSR_ds_; (**f**) RSg_mo_, RSf_t_, and RSf_max_; (**g**) RSτ_m_, RSτgs, and RSτgd; (**h**) RSS_21_ and RSh_21_ at 45 MHz and RSK at 1 GHz The illustrated bias points for intrinsic parameters and RF figures of merits is: V_ds_ = 15 V and V_gs_ = −5 V.

Similarly, to what was done for the DC parameters, the relative sensitivities of the other parameters in Table 1 are calculated using equation 1 and then shown in Figure 11. Because of their low dependence on the temperature, the relative sensitivities of the extrinsic capacitances and inductances are almost nil, as depicted in Figure 11a,b. It can be observed in Figure 11c–e that the relative sensitivities of the extrinsic and intrinsic resistances are positive, reflecting the fact that the resistive contributions increase at higher temperatures. Figure 11f illustrates that unlike the resistances, the transconductance has a negative relative sensitivity, as this parameter is degraded when heating the device.

As illustrated in Figure 11d, the relative sensitivity of C_gs_ is negative, while the relative sensitivities of C_gd_ and C_ds_ are positive. Figure 11g shows that the relative sensitivities of the intrinsic time constants are positive, indicating that they increase when the temperature is raised. This finding implies that the NQS effects occur at lower frequencies when the device is heated. As can be observed in Figure 11f, the relative sensitivities of f_t_ and f_max_ are negative, implying lower operating frequencies at higher temperatures. Figure 11h shows that the relative sensitivities of the magnitude of S_21_ and h_21_ at 45 MHz are negative, in line with the reduction of the transconductance at higher temperatures, while the stability factor (K) shows a positive temperature sensitivity as illustrated at 1 GHz.

**Figure 12:**
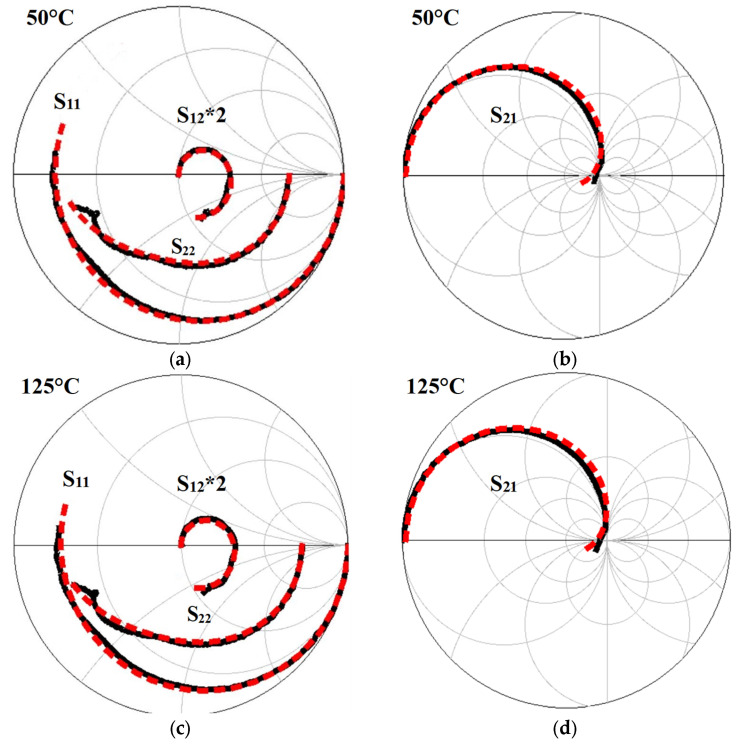
Measured (solid black lines) and simulated (dashed red lines) S-parameters from 45 MHz to 50 GHz for the studied GaN HEMT at 50 °C (**a**,**b**) and 125 °C (**c**,**d**). The illustrated bias point is: V_ds_ = 15 V and V_gs_ = −5 V.

For the tested device, a good agreement between measured and simulated S-parameters was achieved. As an example, Figure 12 depicts the comparison between measurements and S-parameter simulations at two different T_a_ for the tested GaN HEMT at the selected bias condition. The simulations are obtained using the equivalent-circuit model depicted in Figure 8 by means of the commercial microwave simulation software advanced design system (ADS). The small-signal ECPs extracted for different T_a_ from the measured S-parameters are used as inputs to the schematic.

## 4. Conclusions

We have reported an experimental investigation on the impact of the ambient temperature on the DC and microwave performance of a transistor based on an ultra-short 0.15-μm GaN HEMT technology. Measurements have been coupled with an equivalent-circuit model and a sensitivity-based study to assess the thermal effects on device performance over the wide temperature range going from −40 °C to 150 °C. The relative sensitivity was used as the evaluation indicator for this study because it enables investigation of the effects of the ambient temperature on the device performance in a quantitative, systematic, and simple way. The measurement-based findings show that both DC and microwave performance of the studied device are remarkably degraded with increasing temperature.

## Figures and Tables

**Table 1:** Analyzed parameters for the studied GaN HEMTs at 25 °C for the bias condition of V_ds_ = 15 V and V_gs_ = −5 V.

Parameters	Value	Parameters	Value
I_ds_ (mA)	44	C_gs_ (fF)	154
g_m_ (mS)	54	C_gd_ (fF)	28
V_th_ (V)	−6	C_ds_ (fF)	121
V_gm_ (V)	−5.1	R_gs_ (Ω)	1.7
C_pg_ (fF)	50	R_gd_ (Ω)	11.3
C_pd_ (fF)	86	R_ds_ (Ω)	225
L_g_ (pH)	141	g_mo_ (mS)	63
L_s_ (pH)	1.8	τ_m_ (ps)	3.0
L_d_ (pH)	63	τ_gs_ (ps)	1.6
R_g_ (Ω)	2.7	τ_gd_ (ps)	2.0
R_s_ (Ω)	3.0	f_t_ (GHz)	47.3
R_d_ (Ω)	5.8	f_max_ (GHz)	97

## Data Availability

The data presented in this study are available on request from the authors.
